# Application of MRI-guided hysteroscopic one-step resection in preserving the fertility of early endometrial cancer patients

**DOI:** 10.3389/fonc.2025.1597185

**Published:** 2025-08-25

**Authors:** Yi Yu, Lu Zhang, Long Sui, Hongwei Zhang, Limei Chen

**Affiliations:** ^1^ Hysteroscopy Center, Obstetrics and Gynecology Hospital, Fudan University, Shanghai, China; ^2^ Shanghai Key Laboratory of Female Reproductive Endocrine Related Diseases, Shanghai, China

**Keywords:** hysteroscopic one-step resection, magnetic resonance imaging (MRI), endometrial cancer (EC), fertility-preserving treatment, myometrial invasion (MI)

## Abstract

**Objective:**

This study aims to evaluate the role of MRI-guided hysteroscopic one-step precise resection in diagnosing suspected myometrial invasion (MI) of endometrial cancer (EC) in patients desiring fertility preservation and to analyze the impact of suspected MI on the outcomes of fertility-preserving treatments.

**Methods:**

A total of 169 patients with early-stage endometrial cancer who required fertility preservation were enrolled. Among them, 103 cases were ruled out for myometrial invasion by MRI (control group), while 66 cases exhibited suspected myometrial invasion. MRI-guided hysteroscopic one-step resection, which involved the removal of the endometrial lesion, the underlying basal layer, and 3–5 mm of myometrium, was performed for pathological examination. Patients with pathological exclusion of myometrial invasion received fertility-preserving treatment, and their clinical characteristics and treatment outcomes were compared with those of the control group.

**Results:**

Based on the precise diagnosis of MRI-guided hysteroscopic one-step resection, 14 of the 66 patients with suspected myometrial invasion were confirmed by pathology and were included in the MI group for surgical treatment. The remaining 52 patients with no evidence of myometrial invasion were included in the non-MI group and received fertility-preserving treatment. The cumulative complete remission rate in the non-MI group was lower than that in the control group at 6 months (24.9% vs. 59.0%, *P* = 0.021) and 18 months (86.5% vs. 95.1%, *P* = 0.036). The cumulative recurrence rate in the non-MI group was higher than that in the control group after 12 months of follow-up (*P* = 0.037). There was no significant difference in the pregnancy rate between the non-MI group and the control group. There were three cases (25%) of successful pregnancy and full-term delivery in the non-MI group.

**Conclusion:**

MRI-guided hysteroscopic one-step resection can accurately diagnose the presence of myometrial invasion in early endometrial cancer, which helps about 79% of patients preserve their fertility compared with MRI evaluation alone. Hysteroscopic resection of endometrial lesions, high-dose progesterone treatment, and follow-up are important for the successful fertility-preserving treatment of patients with early endometrial cancer.

## Introduction

As one of the most common gynecological malignancies that affect women’s life and health, endometrial cancer (EC) is prevalent among postmenopausal women, but its incidence has shown a trend of younger age, with about 4.5% of EC cases occurring in young women under the age of 40 (1, 2). Although hysterectomy is the standard treatment for endometrial cancer, it comes at the cost of permanent loss of fertility for young patients. Therefore, how to reverse endometrial lesions while preserving the fertility of young patients under the premise of ensuring life safety has become a focus of attention. According to the ESGO/ESHRE/ESGE guidelines and the practical guideline on the fertility-sparing treatment of patients with endometrial carcinoma and atypical endometrial hyperplasia, G1 and IA endometrial cancer cases limited to the endometrial layer meet the criteria for fertility-preserving treatment (3, 4). Endometrial cancer confined to the endometrial layer responds well to conservative treatment with progesterone-based therapy, with high complete response rates and low recurrence rates (5, 6). Therefore, accurate judgment of whether there is myometrial invasion (MI) in endometrial cancer is crucial to determining whether a patient can receive fertility-preserving treatment and estimating the effect of progesterone therapy.

Magnetic resonance imaging (MRI) is an effective tool to diagnose myometrial invasion in patients with endometrial cancer, with sensitivity and specificity of 80%–93% and 74%–90%, respectively (7–9). However, MRI has a certain rate of false positives and false negatives, and its accuracy to diagnose myometrial invasion is poor. Sometimes the abnormal imaging manifestations of myometrial invasion are not significant, and only a slight ambiguity and poor continuity of the endometrial basal layer can be observed (10, 11). In this case, it can only be assessed as “suspected myometrial invasion”. Therefore, for young patients with suspected myometrial invasion on MRI, how to accurately determine whether there is myometrial invasion is an important issue to determine whether fertility-preserving treatment can be performed.

Hysteroscopic electro-resection of endometrial lesion, basal layer, and myometrial layer and pathological examination provided an effective solution for the diagnosis of endometrial carcinoma myometrial invasion (12, 13). In 2010, Mazzon (14) first reported six cases of patients with endometrial cancer who underwent hysteroscopic resection of the lesion, during which both the endometrial tissue around the tumor and the myometrium under the tumor were removed (three-step resection). For patients with negative resection margins and no evidence of myometrial invasion, progesterone therapy was started after surgery. Laurelli (15) reported 14 cases of patients with endometrial cancer who underwent hysteroscopic electro-resection, including the lesion and the myometrium tissue (two-step resection), and the postoperative treatment for patients in whom myometrium invasion was excluded. However, both of the two methods underwent fractional electrosurgical resection, which caused tissue fragmentation and affected the pathological diagnosis. The thermal effect of electrosurgical resection would cause permanent damage to the endometrium and affect the pregnancy outcome after complete remission of endometrial cancer.

In addition, we further evaluated the fertility-preserving effect of patients with suspected myometrial invasion by MRI but who were pathologically excluded after hysteroscopic one-step resection compared with patients without signs of myometrial invasion by MRI and evaluated the impact of suspected myometrial invasion on fertility-preserving effect so as to provide guidance for the clinical diagnosis and treatment of these cases.

## Methods

### Patients

Patients with early endometrial cancer who were admitted to the Obstetrics and Gynecology Hospital Affiliated to Fudan University from August 2017 to August 2023 and hoped to preserve their fertility were enrolled. All patients were prospectively registered in the database and received standardized assessments and treatments during the study. This study was approved by the Ethics Committee of the Obstetrics and Gynecology Hospital Affiliated to Fudan University (no. 2018-61).

The inclusion criteria were as follows (1): have been fully informed that fertility-preserving therapy is not the standard treatment for endometrial cancer and the related risk, have agreed for their clinical data to be used for scientific research, and have signed the informed consent (2); age<45 years old (3); have a strong desire to preserve their fertility (4); the pathological diagnosis of endometrial was G1 after the first curettage or hysteroscopy (5); there was no evidence of myometrial invasion of endometrial carcinoma as evaluated by pelvic MRI and no evidence of cervical invasion or extrauterine metastasis; and (6) there was no contraindication of progesterone therapy.

The exclusion criteria were as follows (1): severe infection or serious chronic diseases (such as heart, liver, lung, or renal insufficiency) (2), recurrent endometrial carcinoma, and (3) history of other malignant tumors.

### MRI evaluation and localization of endometrial cancer

**Figure 1:**
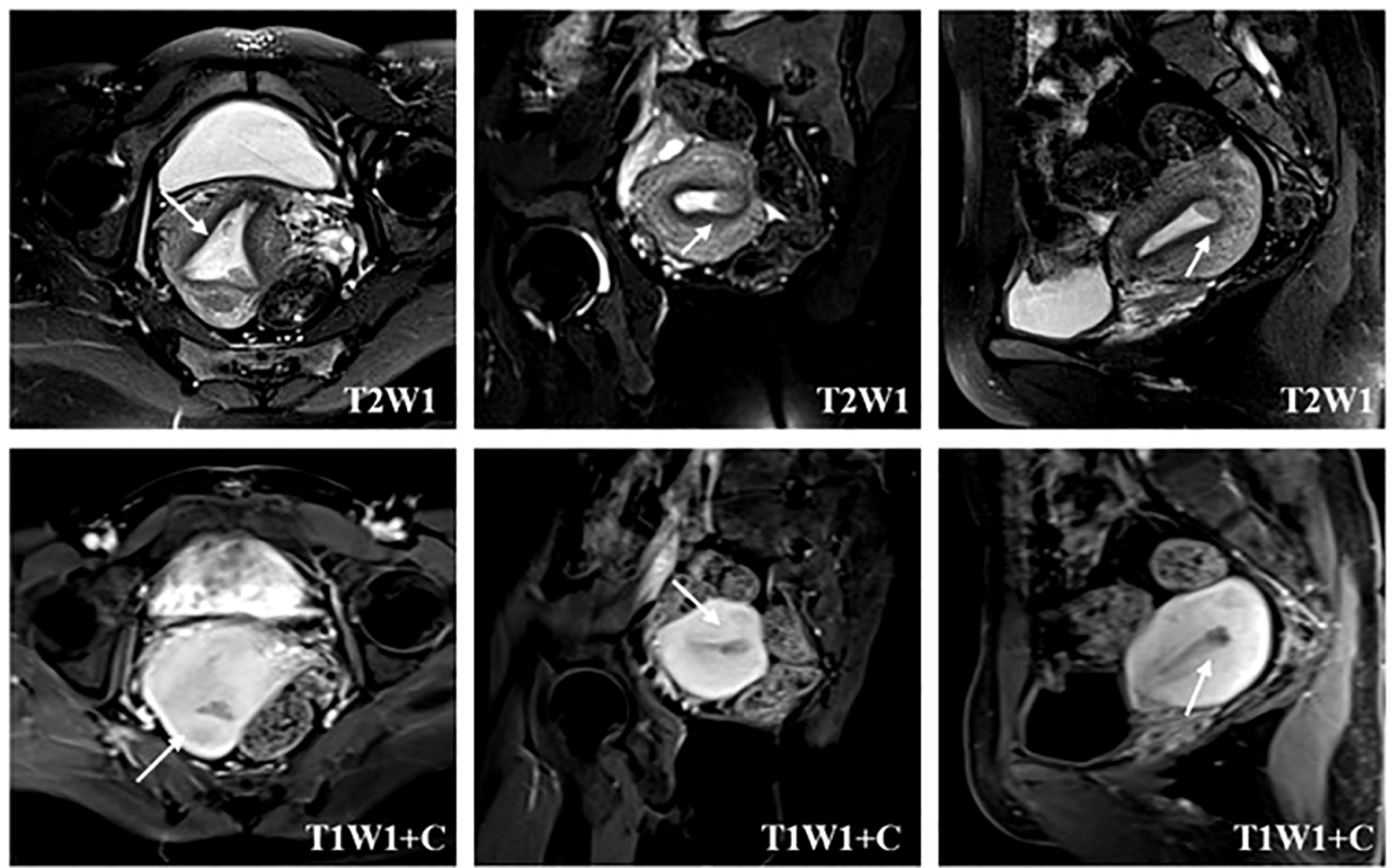
Localization of suspected myometrial invasion on MRI. The T2-weighted image reveals the heterogeneous signal intensity of the endometrial–myometrial junctional zone (arrow) in the lower left uterine horn. The contrast-enhanced T1-weighted image depicts an interrupted enhancing line (arrow) under the endometrium, suggesting myometrial invasion.

The patient underwent pelvic enhanced MRI examination. Intravenous contrast agent (0.2 mL/kg body weight GD DTPA) was used for T1WI+C imaging. MRI images were read independently by two experienced radiologists and reviewed by two experienced senior radiologists. The imaging evidence of MRI diagnosis of myometrial invasion of endometrial carcinoma was that the endometrium–myometrium junction was completely invisible or irregular and sharp. If MRI only showed local blurring or poor continuity of the endometrium–myometrium junction, it was diagnosed as suspicious myometrial invasion. For the suspicious myometrial invasion found on imaging, the location and scope of the suspected lesions in the uterine cavity can be located by the sagittal, coronal, and transverse images of pelvic MRI so as to perform one-step hysteroscopic resection to determine the exact depth of invasion of endometrial cancer (**Figure 1**).

### MRI-guided one-step hysteroscopic resection

The suspected lesion was performed by one-step hysteroscopic resection, and the underlying basal layer and muscular layer were resected together, and the myometrial invasion was accurately judged by pathological diagnosis. For suspected myometrial invasive lesions, the three-step resection method reported in the previous literature was not adopted, but the one-step resection method was used. The specific steps are detailed below.

The shape, color, and thickness of the endometrium were evaluated under hysteroscopy, and the lesions were observed and recorded. According to the MRI imaging and intraoperative findings, the lesions at different locations were sampled at fixed points. Endometrial lesions without myometrial invasion were removed with micro-scissors. The lesions suspected of myometrial invasion were accurately located under MRI-guided hysteroscopy, and an electrosurgical excision ring with a width of 5 mm and a depth of 5 mm was used to perform one-step resection: that is, the endometrial lesions, the underlying basal layer, and the myometrium with a depth of 3–5 mm were resected at the same time. If the thickness of the lesion was thin (≤2 mm), it was directly cut to the superficial myometrium 3–5 mm by one-step resection. If the thickness of the lesion was thicker (>2 mm), the lesion should be bluntly removed by using the electrosurgical ring to ensure the depth of subsequent resection, and then the superficial myometrium 3–5 mm was resected by one-step electrosurgical method. If the lesion was large, multiple resections can be performed if necessary until the suspected myometrial invasive lesion, including 2 to 3 mm endometrium around the lesion, was completely removed. The residual lesions were removed with micro-scissors to reduce electrical damage to the surrounding endometrium and prevent uterine adhesions. The pathology results confirmed that endometrial cancer patients with myometrial invasion underwent hysterectomy; patients with pathological exclusion of myometrial invasion received fertility-preserving therapy.

### Efficacy evaluation of the fertility-preserving treatment

For patients with early endometrial cancer who have no evidence of myometrial invasion, the first-line treatment was megestrol (160 mg/time) once a day if BMI <30. If it was ineffective or the patient had a history of breast cancer or with BMI ≥ 30, the second-line treatment was a combination of GnRH and letrozole. Some patients were given metformin (0.5 g/time) three times a day or had levonorgestrel intrauterine contraceptive system (LNG-IUS) concurrently. Patients with high blood lipids should take rosuvastatin. All patients received hysteroscopic evaluation every 3 months during the fertility-preserving treatment and pelvic MRI every 6 months to evaluate the effectiveness of the treatment and disease progression.

Complete response (CR) is defined as no abnormal changes in pathological results, that is, two consecutive hysteroscopic pathological results suggest secretory endometrium/proliferative endometrium/endometrial hyperplasia disorder. Partial response (PR) is defined as the improvement of pathological results, that is, two consecutive hysteroscopic pathological results suggest simple hyperplasia/complex hyperplasia/atypical hyperplasia. No response (NR) is defined as the same pathological results as before the treatment. Recurrence is defined as any abnormal endometrium indicated by the pathological results after the patient has achieved CR during the follow-up period.

After the patients who receive fertility-preserving treatment achieve CR, they will continue with consolidation treatment for 2 to 3 months. We encourage patients with CR to actively receive assisted reproductive therapy. For CR patients without pregnancy plans in the near future, low-dose progesterone, oral contraceptives, or LNG-IUS is recommended to prevent recurrence. Follow-up evaluations for CR patients are conducted every 3–6 months, utilizing ultrasound and endometrial sampling biopsies to evaluate the endometrium. If any abnormalities are detected, hysteroscopy will be performed. For patients with NR after more than 6 months of treatment or with disease progression during the fertility-preserving treatment, hysterectomy is recommended.

### Statistical analysis

Complete remission time was defined as the time from the progesterone treatment to the first pathological diagnosis of CR. Recurrence time was defined as the time from the first pathological diagnosis of CR to recurrence.

All statistical analyses were performed using SPSS 20.0. Age, BMI, complete remission time, follow-up time, and recurrence time were expressed in median. Lesion size (>2 cm or ≤2 cm) and pregnancy were expressed as proportions. Student’s test and Mann–Whitney *U*-test were used to compare the intra-group differences, while chi-square test was utilized to compare frequency distributions. Kaplan–Meier method was applied to describe treatment and recurrence time. Log-rank test was used to evaluate differences between groups. *P <*0.05 was considered statistically significant.

## Results

### Clinical characteristics of patients

**Figure 2:**
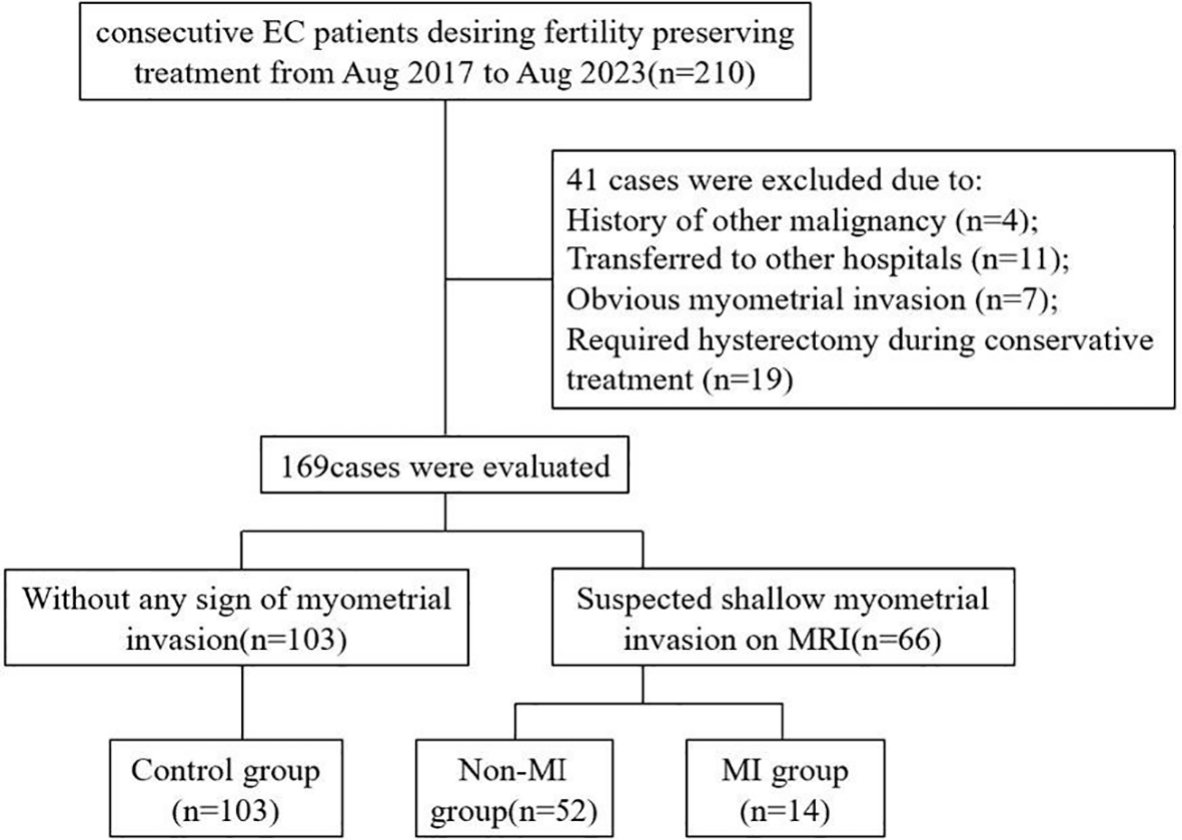
Flowchart of the screening of the study population.

A total of 210 patients with early endometrial cancer who wanted to preserve fertility in the Obstetrics and Gynecology Hospital of Fudan University were enrolled in this study from August 2017 to August 2023. Among them, 41 patients were excluded according to the exclusion criteria, and 169 patients met the inclusion criteria and were recruited into the study, including 66 patients with suspected superficial myometrial invasion on MRI and 103 patients without any evidence of myometrial invasion (control group). A total of 66 patients with suspected myometrial invasion underwent hysteroscopy and MRI-guided one-step resection. Myometrial invasion was confirmed according to postoperative pathology. Among them, 14 patients with myometrial invasion confirmed by postoperative pathology were included in the myometrial invasion group (MI group), while the 52 patients exhibited no evidence of myometrial invasion and were included in the non-MI group (**Figure 2**).

The clinical characteristics of patients in the non-MI group and the control group are shown in **Table 1**. There were no statistically significant differences in age, BMI, and fertility history between the two groups. However, patients with suspected myometrial invasion on MRI tended to have larger lesions compared to those without myometrial invasion: 43 patients (41.2%) in the control group had lesion diameter >2 cm, while 37 patients (71.2%) in the non-MI group had lesion diameter >2 cm (*P* = 0.023). Both the non-MI group and the control group received fertility-preserving therapy, and the median follow-up time was 21 (12–46) months and 22 (10–48) months, respectively. The median follow-up time after CR was 12 (3–21) months in the non-MI group and 13.5 (3–30) months in the control group, respectively.

**Table 1:** Clinical characteristics of patients with endometrial carcinoma in the control group and the non-MI group [*n* (%) or M (range)].

Variable	Non-MI group	Control group	*P*
Patient number (*n*)	52	103	–
Age (years)	31 (24–42)	32 (25–44)	0.36
BMI (kg/m^2^)	28.4 (24.9-34.7)	26.8 (19.8-32.5)	0.17
Nulliparous	50 (96.2)	98 (95.1)	0.54
Lesion size (cm)			**0.023**
≤2	15 (28.8)	60 (58.3)	–
>2	37 (71.2)	43 (41.2)	–
Progestin therapy			
MA	5 (9.6)	26 (25.2)	–
MA + metformin	14 (26.9)	17 (16.5)	–
MA + LNG-IUS	5 (9.6)	25 (24.3)	–
MA + metformin + LNG-IUS	4 (7.7)	13 (12.6)	–
MA + metformin + rosuvastatin	9 (17.3)	11 (10.7)	–
GnRHa + letrozole therapy	15 (28.8)	11 (10.7)	–
18-month cumulative CR	45 (86.5)	98 (95.1)	**0.036**
Median treatment duration to CR (months)	11 (3–24)	7 (1–24)	0.079
Median follow-up duration (months)	21 (12–46)	22 (10–48)	0.064
Median follow-up after CR (months)	12 (3–21)	13.5 (3–30)	0.092
12-month cumulative relapse	8.9 (4/45)	0 (0/98)	**0.037**
Pregnancy rate^a^	25 (3/12)	21 (5/24)	–

BMI, body mass index; MI, myometrial invasion; LNG-IUS, levonorgestrel intrauterine device.

a Pregnant patients among patients who achieved CR and actively prepared for pregnancy.

Values in bold indicate statistically significant results between the non-MI group and control group with a preset threshold of p < 0.05.

### MRI-guided hysteroscopic one-step resection

**Figure 3:**
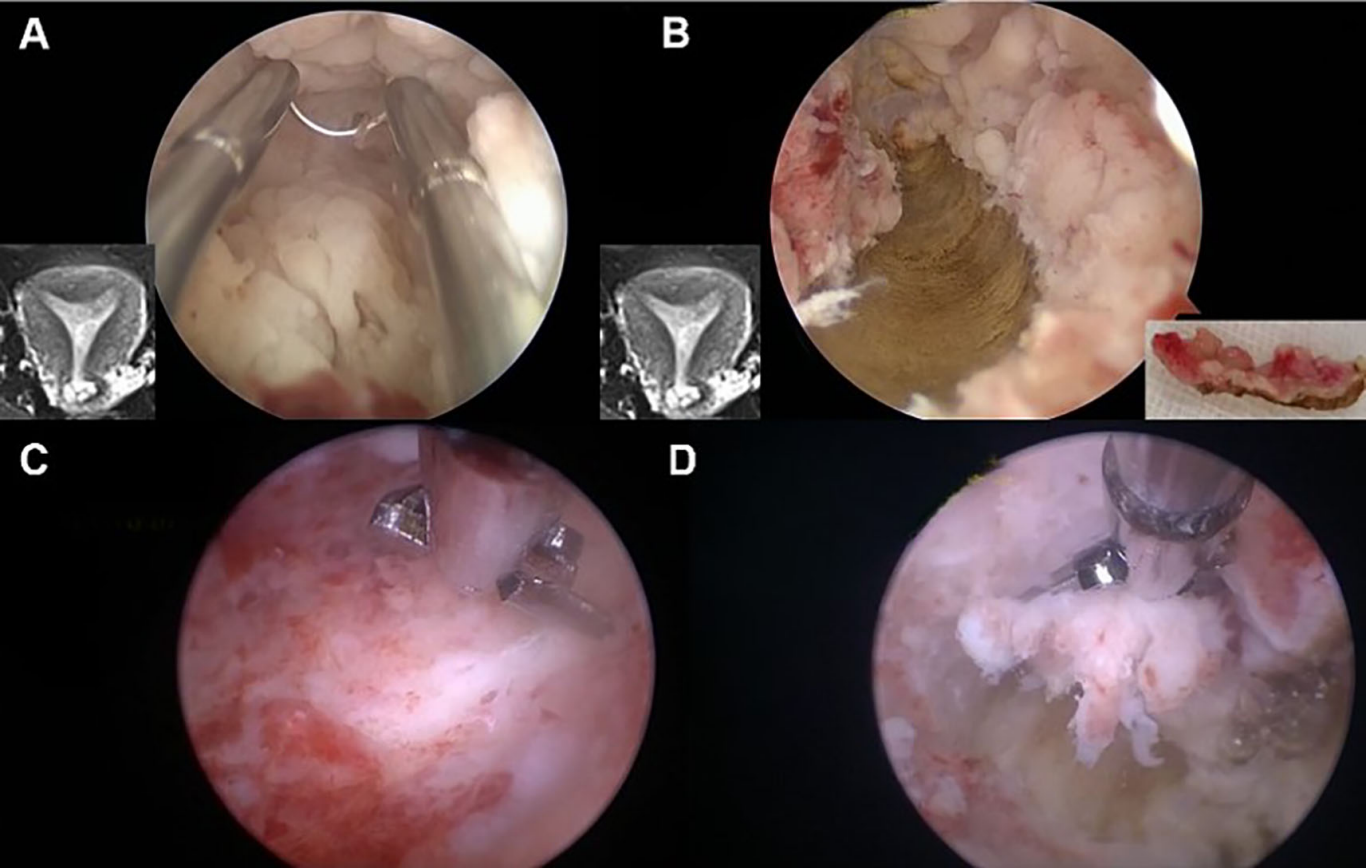
Hysteroscopic one-step resection in early EC patients. **(A)** The lesions suspected of muscular involvement were accurately located under hysteroscopy according to MRI. **(B)** During electrosurgical resection, “one continuous resection” was applied to resect the myometrium, with the endometrium connected to the myometrium. **(C, D)** The residual lesions were removed with scissors to reduce electrical damage to the endometrium and prevent uterine adhesions.

Take the 29-year-old girl shown in **Figure 3** for example. She was married but had no children, and her body mass index (BMI) is 34.1. She had abnormal uterine bleeding (AUB) for half a year and then undertook diagnostic curettage in the local hospital. The pathology results showed that there was endometrioid adenocarcinoma. This patient was referred to our hospital due to her strong desire to preserve her fertility. The MRI showed an abnormal signal in the left cornua of the uterine cavity, with a size of 1.6 cm × 0.9 cm × 0.6 cm, and the junction zone boundary was not clear. Myometrial invasion of the lesion cannot be completely excluded. Therefore, hysteroscopy was performed to assess potential myometrial involvement and the feasibility of fertility preservation. The pathology results after hysteroscopy showed small-scale endometrial carcinoma limited to the fundus endometrium, along with diffuse atypical hyperplasia and normal myometrium. Thus, we proceed with fertility-preserving treatment for her.

### Clinical parameters of fertility-preserving treatment

We further investigate if whether, for endometrial cancer patients with MRI-suspected myometrial invasion and pathological results that excluded myometrial invasion, the efficacy of fertility-preserving therapy will be comparable to that of patients without any evidence of myometrial invasion. The cumulative CR rates of the non-MI group and the reference group at 3, 6, 12, 18, and 24 months after the fertility-preserving treatment are shown in **Table 2**.

**Table 2:** Cumulative complete response rate of patients with treatment of fertility preservation in the control group and the non-MI group (%).

CR rate	Non-MI group	Control group	*P*
3 months	9.7	23.8	0.37
6 months	24.9	59.0	**0.021**
12 months	61.3	74.8	0.69
18 months	86.5	95.1	**0.036**
24 months	88.4	95.1	**0.029**

MI, myometrial invasion; CR, complete response; 3 months CR rate, cumulative CR rate at the third month of follow-up.

Values in bold indicate statistically significant results between the non-MI group and control group with a preset threshold of p < 0.05.

After 3 months of treatment, there was no significant difference in the cumulative CR rate between the two groups (9.7% vs. 23.8%, *P* = 0.37). However, at the 6th month of treatment, the cumulative CR rate of the non-MI group was lower than that of the control group (24.9% vs. 59.0%, *P* = 0.021). With the extension of treatment time, the cumulative CR rate of the non-MI group gradually increased, and there was no significant difference between the two groups at the 12th month of treatment. After 18 months of treatment, the cumulative CR rate in the non-MI group was lower than that in the control group (86.5% vs. 95.1%, *P* = 0.036). The median time to CR in the non-MI group and the control group was 11 (3–24) months and 7 (1–24) months, respectively, with no significant difference (*P* = 0.079).

No serious adverse events related to drug treatment or hysteroscopy, such as uterine perforation, thromboembolism, severe liver and kidney dysfunction, or severe infection, were found in either group.

### Disease recurrence and pregnancy

Patients receiving fertility-preserving treatment entered the follow-up period after achieving CR. A total of 45 patients in the non-MI group achieved CR, and 98 patients in the control group achieved CR. The median follow-up time after CR was 12 (3–21) months in the non-MI group and 13.5 (3–30) months in the control group. During the follow-up period after CR, there were four cases of recurrence in the non-MI group and no recurrence in the control group. According to the Kaplan–Meier curve, the cumulative recurrence rate at 12 months after CR in the non-MI group was 8.9%, which was higher than that in the control group (*p* = 0.037).

Among the patients in the non-MI group who achieved complete remission, 12 patients planned pregnancies, of which three (25%) successfully got pregnant. One case was conceived naturally, and two cases were conceived through *in vitro* fertilization. These three cases were all terminated pregnancy by cesarean section after full-term pregnancy without obstetric complications. Of the patients in the control group, 24 had near-term fertility plans after CR, and five (21%) had a successful pregnancy, respectively. All five cases were conceived through *in vitro* fertilization. Four cases had full-term delivery, one case had premature birth, and one case had adherent placenta.

## Discussion

Endometrial cancer is the most common tumor in gynecology, and its incidence among women of reproductive age has been on the rise in recent years. How to treat endometrial cancer patients of reproductive age who have not yet been pregnant and still have good fertility is a question worthy of exploration. The latest research evidence indicates that well-differentiated, early-stage endometrial adenocarcinoma confined to the endometrium without myometrial invasion constitutes the main criterion for fertility preservation in young patients (16–18). These patients respond well to progesterone and have a low recurrence rate. Once endometrial cancer invading on the myometrium will affect the efficacy of progesterone treatment, this results in an increased risk of disease progression and recurrence (5). Therefore, accurate diagnosis of endometrial cancer myometrial invasion prior to fertility-preserving treatment is crucial for young patients who want to preserve their fertility. As a common method to evaluate myometrial invasion of endometrial cancer, MRI demonstrates high diagnostic sensitivity and specificity (19, 20). However, its effectiveness in accurately diagnosing superficial myometrial invasion is unsatisfactory. Sometimes the endometrial–myometrial junctional band is blurred in the images, providing insufficient evidence to confirm myometrial invasion. In 2018, Sakane et al. evaluated the diagnostic capability of MRI for myometrial invasion in premenopausal women with endometrial cancer and found that MRI exhibited poor diagnostic efficacy for superficial myometrial invasion with an invasion depth of ≤3 mm (10).

The limitations of MRI in the diagnosis of myometrial invasion of endometrial cancer may be due to the following reasons (21, 22) (1): Many patients underwent MRI shortly after endometrial biopsy. Intraoperative endometrial curettage may damage the endometrial basal layer, resulting in blurred or interrupted endometrial–myometrial junctions on MRI images, which are similar to the imaging characteristics of myometrial invasion, thus complicating the diagnosis (2). The signal intensity of the uterus in premenopausal women was high on MRI images, making it challenging to differentiate the lesion location from normal myometrium (3). The signal intensity of the endometrial–myometrial junction and the sub-endometrial enhancement line fluctuates periodically due to hormonal influences, resulting in different MRI diagnostic capabilities for myometrial invasion (4). Concurrent conditions such as uterine fibroids, adenomyosis, and atypical polypoid adenomyoma can also interfere with MRI diagnosis of myometrial invasion in endometrial cancer. The ambiguity in diagnosing myometrial invasion by MRI can significantly impact the formulation and implementation of fertility-preserving treatment strategies for endometrial cancer.

In order to make up for the defect of MRI in the diagnosis of myometrial invasion of endometrial cancer and improve the diagnostic accuracy, we performed the MRI-guided one-step hysteroscopic resection for the accurate diagnosis of early myometrial invasion of endometrial cancer. MRI can accurately assess the size and location of the tumor as well as the suspected myometrial invasion. Then, the endometrium, basal layer, and myometrium of the suspected myometrial invasion site were resected by hysteroscopy. For lesions in the MRI scans that indicate suspected myometrial invasion, the two-step and three-step hysteroscopic electroresection methods reported in previous literature involve the separate removal of the endometrium, basal layer, and myometrial layer. This approach loses the reference for the interrelationship among the three layers, potentially leading to an inability to accurately determine whether there is myometrial invasion. Moreover, multiple electro-resections can cause electrical damage to the removed tissues, resulting in severe destruction of the microscopic tissue structure and affecting the effective pathological diagnosis. The extensive resection and the thermal effect of electroresection will cause permanent damage to the endometrium. The divided electroresection method prolongs the surgical time and increases the occurrence of surgical complications such as intrauterine adhesions (23, 24).

Compared with the traditional two-step and three-step hysteroscopic resection, our one-step resection can ensure the integrity of the excision tissue and help the pathologist to better judge the myometrial invasion. Because of the reduction of the time of electrosurgical resection, it can protect the endometrium, especially the basal layer, and prevent uterine adhesions and thinning of the endometrium, thus improving the pregnancy outcome. In order to minimize endometrial damage and the incidence of intrauterine adhesions, a micro-scissor was used to cut off small or residual lesions to reduce thermal damage. In recent years, a new type of cold-knife operating system, tissue removal devices, has offered advantages such as precise cutting and preservation of the functional layer of the endometrium as it relies on mechanical resection without the use of high-frequency electric current (25, 26). However, due to the large number of surgeries in our center and the limited availability of tissue removal devices, micro-scissors were used for all cold-knife procedures in this study. In future research, prospective studies will be conducted using tissue removal devices.

Our study showed that if MRI showed suspected myometrial invasion in endometrial carcinoma, the patients subsequently received MRI-guided hysteroscopic precise positioning one-step electrosurgical resection; 78% of patients were excluded from myometrial invasion by postoperative pathology and obtained opportunities for fertility-preserving treatment. Hysteroscopic precise one-step resection can make up for the limitations of MRI in diagnosing superficial myometrial invasion and provide a novel diagnostic approach for clinical evaluation of myometrial invasion in patients with early endometrial cancer.

We observed that when MRI indicated suspicious myometrial invasion in endometrial cancer, the outcomes of fertility-preserving treatment were significantly worse than that for patients without any sign of myometrial invasion: the complete remission rates at 6 and 18 months were lower, and the cumulative recurrence rate within 12 months after complete remission was higher, even when the pathology results after hysteroscopic one-step resection ruled out myometrial invasion. The possible explanations for this result are as follows: the patients in the control group did not have any evidence of myometrial invasion on MRI before the fertility-preserving treatment, and the lesions were limited to the endometrium, which had a better response to progesterone and was easier to be removed by hysteroscopy. In contrast, in the non-MI group, MRI showed suspected myometrial invasion, and the pathology results also revealed that the endometrial cancer lesions approached or reached the endometrial basal layer. Such lesions had a poorer response to drug treatment and were not easily removed mechanically, resulting in poor subsequent fertility-preserving treatment outcomes. Moreover, although no evidence of myometrial invasion was found in the pathological examination of the non-MI group, the possibility of myometrial invasion that was not detected by hysteroscopy cannot be excluded. Given these findings, caution is warranted in clinical diagnosis and treatment. For patients with endometrial cancer (stage I, grade 1) and suspected myometrial invasion on MRI, clinicians should inform patients of the relatively poor prognosis and increased risk of recurrence associated with continued fertility-preserving treatment, even if the hysteroscopic resection pathology excludes myometrial invasion. In addition, it is suggested that patients should have ultrasound examination every 3 months and pelvic MRI every 6 months during treatment so as to identify myometrial invasion or extrauterine metastasis early.

Some patients with recurrent endometrial cancer during the course of conservative treatment still maintain a strong desire for fertility and opt to undergo another round of conservative management. Studies have indicated that the complete remission rate associated with retreatment is relatively high, with the recurrence rate remaining within an acceptable range. The treatment methods primarily involve high-dose oral progestogens in combination with metformin, gonadotropin-releasing hormone agonists, and hysteroscopic resection, among other adjunctive therapies, to enhance therapeutic outcomes. Individualized treatment strategies should be developed according to each patient’s specific clinical profile (27).

One limitation of this study is that the sample size for follow-up pregnancy outcomes is relatively small. The reason for the small sample size is that there were not many patients suspected of infiltration on MRI. For these patients, they need to undergo comprehensive treatment and achieve complete remission twice before entering into preconception. However, some of these patients are still unmarried or have no fertility planning yet, so the sample size of the pregnancy rate is relatively low. Our research will continue, and there is hope to increase the sample size in the future. Achieving pregnancy after complete remission, implementing weight reduction measures, and undergoing maintenance therapy are recognized as protective factors against disease recurrence. Future research should prioritize the exploration of precision treatment approaches guided by molecular subtyping and the identification of biomarkers with predictive significance, thereby enabling the formulation of more tailored and precise therapeutic plans for patients (28, 29).

## Conclusion

MRI-guided hysteroscopic precise positioning one-step resection is an effective method for diagnosing suspected myometrial invasion of early endometrial cancer. This diagnostic method helps 79% of patients with suspected myometrial invasion by MRI imaging to preserve their fertility. MRI suggesting suspected myometrial invasion is associated with poor outcomes of fertility-preserving treatment in young patients. Therefore, for such cases, precise diagnosis and treatment should be carried out. Under the premise of ensuring the patient’s life safety, efforts should be made to provide fertility-preserving treatment for the patient as much as possible.

## Data Availability

The original contributions presented in the study are included in the article/supplementary material. Further inquiries can be directed to the corresponding author.
